# Management of life-threatening hemoptysis

**DOI:** 10.1186/s40560-020-00441-8

**Published:** 2020-04-05

**Authors:** Hasmeena Kathuria, Helen M. Hollingsworth, Rajendran Vilvendhan, Christine Reardon

**Affiliations:** 1 The Pulmonary Center, Boston University School of Medicine, 72 E. Concord St R304, Boston, MA 02118 USA; 2 Interventional Radiology, Department of Radiology, Boston Medical Center, Boston, MA USA

**Keywords:** Massive hemoptysis, Life-threatening hemoptysis, Bronchial artery embolization (BAE), Bronchoscopy

## Abstract

It is estimated that 5–14% of patients presenting with hemoptysis will have life-threatening hemoptysis, with a reported mortality rate between 9 and 38%. This manuscript provides a comprehensive literature review on life-threatening hemoptysis, including the etiology and mechanisms, initial stabilization, and management of patients. There is no consensus on the optimal diagnostic approach to life-threatening hemoptysis, so we present a practical approach to utilizing chest radiography, computed tomography, and bronchoscopy, alone or in combination, to localize the bleeding site depending on patient stability. The role of angiography and embolization as well as bronchoscopic and surgical techniques for the management of life-threatening hemoptysis is reviewed. Through case presentation and flow diagram, an overview is provided on how to systematically evaluate and treat the bronchial arteries, which are responsible for hemoptysis in 90% of cases. Treatment options for recurrent hemoptysis and definitive management are discussed, highlighting the role of bronchial artery embolization for recurrent hemoptysis.

## Background

It is estimated that 5–14% of patients presenting with hemoptysis will have life-threatening hemoptysis [1–3]. Life-threatening hemoptysis, also called massive hemoptysis, has been variably defined based upon criteria such as the volume per hour of bleeding, the total volume of bleeding per 24 h, or the presence of abnormal gas exchange or hemodynamic instability [1, 2, 4]. No consensus has been determined, but in general, bleeding rates > 100 mL/h or total volumes > 500 mL in 24 h are considered life-threatening hemoptysis [1, 4]. Depending upon the patient’s underlying cardiopulmonary status, smaller volumes (50 mL) of hemoptysis may be life-threatening [5].

The reported mortality rate for life-threatening hemoptysis is between 9 and 38% [6]. Several factors have been identified that predict a poor outcome in patients experiencing life-threatening hemoptysis. These include a rapid rate of bleeding characterized as at least 100 mL within a 24-h period, aspiration of blood into the contralateral lung, or life-threatening bleeding requiring single-lung ventilation [2, 3, 7]. A retrospective cohort study of patients with severe hemoptysis reported risk factors for in-hospital mortality that included the presence of multilobar opacities, the need for mechanical ventilation, involvement of the pulmonary artery, and a diagnosis of cancer, aspergillosis, or chronic alcoholism [8].

## Etiologies of hemoptysis

The literature on the etiology of life-threatening hemoptysis is largely based on retrospective, single-centered studies from diverse geographic patient populations. Understanding these limitations in sample size and selection bias, the most common causes of life-threatening hemoptysis include bronchiectasis from infectious and non-infectious etiologies, bronchogenic carcinoma, and depending on geographical location, various lung infections such as tuberculosis (TB) [1, 4, 5, 9–12]. In an observational, multi-center study in Italy, malignancies and bronchiectasis were the leading causes of moderate (20–500 mL in 24 h) and severe (> 500 mL in 24 h) hemoptysis [13]. Additional etiologies described in the literature include mycetomas, necrotizing pneumonia, and cryptogenic hemoptysis [1, 4, 5, 9–12]. Table 1 outlines the spectrum of etiologies of life-threatening hemoptysis.

**Table 1:** Etiologies of life-threatening hemoptysis

**Intrinsic pulmonary parenchymal disease**	
Bronchiectasis	Sarcoidosis, cystic fibrosis, tuberculosis, nontuberculous mycobacterial, fungal
Pulmonary infections	Tuberculosis, fungal, necrotizing pneumonia, mycetoma, lung abscess, parasitic infection (*Paragonimus westermani*)
Pulmonary malignancy	Bronchogenic carcinoma, endobronchial metastases, bronchial adenoma
Pulmonary vascular	Non-iatrogenic: arteriovenous malformation, subepithelial bronchial artery (Dieulafoy), aortic aneurysm with erosion, pulmonary embolism (septic or thrombotic) Iatrogenic injuries: Pulmonary artery injury from pulmonary artery catheter, aortobronchial fistula due to aortic graft or stent, airway stent, biopsy complications from bronchoscopic procedures
Pulmonary trauma	Penetrating chest injury, blunt force chest injury
**Medication and toxins**	Cocaine, bevacizumab, anticoagulants and antiplatelet medications, nitrogen dioxide
**Collagen vascular diseases involving the lung**	Systemic lupus erythematosus, granulomatosis with polyangiitis or other vasculitides, anti-glomerular basement membrane disease, idiopathic hemosiderosis, amyloidosis, Behcet disease
**Cardiovascular diseases**	Pulmonary edema from heart failure, mitral stenosis, tricuspid endocarditis, congenital heart disease
**Bleeding disorders**	Disseminated intravascular coagulation, thrombocytopenia, von Willebrand disease, platelet dysfunction

## Mechanism of rupture

The high pressure bronchial artery circulation is the source of hemoptysis in 90% of cases. The bronchial arteries most commonly originate from the descending aorta at the level of the T5–T6 vertebral bodies. Anomalous bronchial arteries (those that arise outside of the area between the T5 and T6 vertebrae) can arise from the aortic arch, subclavian artery, brachiocephalic trunk, intercostal arteries, thyrocervical and costocervical trunk, internal mammary artery, pericardiophrenic and inferior phrenic artery, abdominal aorta, and coronary arteries (reported prevalence is 8–35%) [14]. The bronchial arteries, subject to systemic blood pressure, are altered in chronic infectious or inflammatory lung diseases, leading to an increased risk of bleeding [6, 15]. Chronic or recurrent infection and inflammation cause bronchial arteries to become dilated and tortuous. With infection and/or inflammation, the normal vascular anastomoses between bronchial arteries and pulmonary vessels become more prominent leading to greater blood flow through the dilated bronchial arteries [15]. In addition, new and collateral vessels, promoted by the release of angiogenic growth factors such as vascular endothelial growth factor, have thin walls and are prone to rupture [16].

Other vascular sources of hemoptysis include the non-bronchial systemic collaterals that are recruited secondary to chronic lung inflammation. Non-bronchial systemic arteries are the source of bleeding in 5% of cases. Non-bronchial systemic collaterals originating from the descending thoracic aorta, intercostals, subclavian, brachiocephalic, internal mammary, and axillary arteries differ from anomalous bronchial arteries in that their course is not parallel to the bronchi [6, 17–20].

In approximately 5% of cases, the pulmonary arteries are the source of hemoptysis (e.g., rupture of a Rasmussen’s aneurysm, which is derived from the pulmonary arterial circulation). Rarely, the aorta (ruptured aortic aneurysm or an aortobronchial fistula) and the bronchial and pulmonary veins can lead to hemoptysis.

## Case presentation

**Fig. 1:**
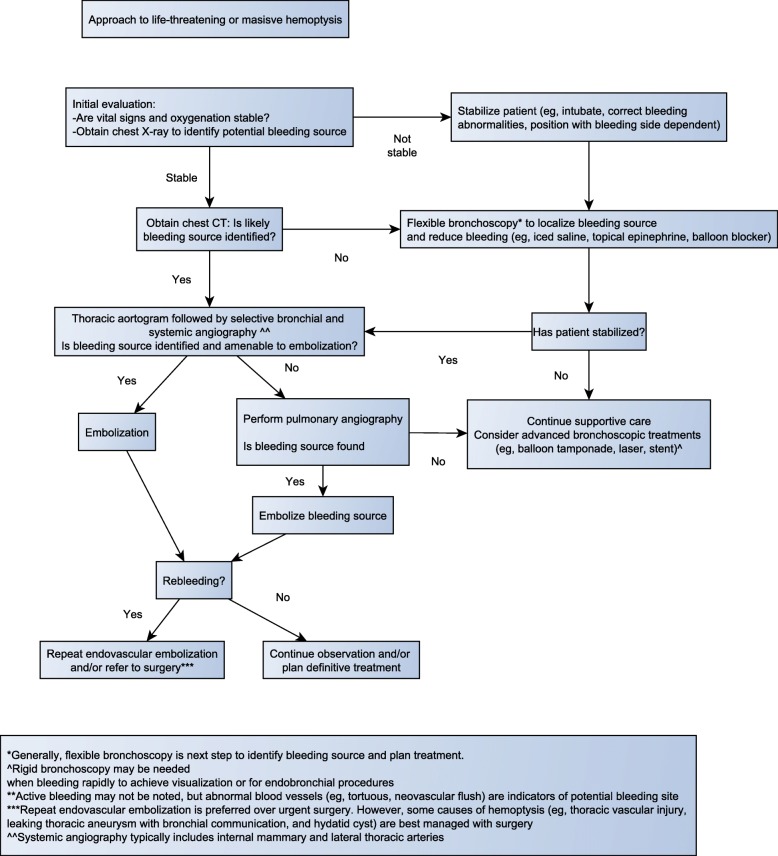
Approach to life-threatening hemoptysis

The following case and the discussion that follows illustrate the initial evaluation and management and the diagnostic and therapeutic considerations in patients presenting with life-threatening hemoptysis (Fig. 1).

**Fig. 2:**
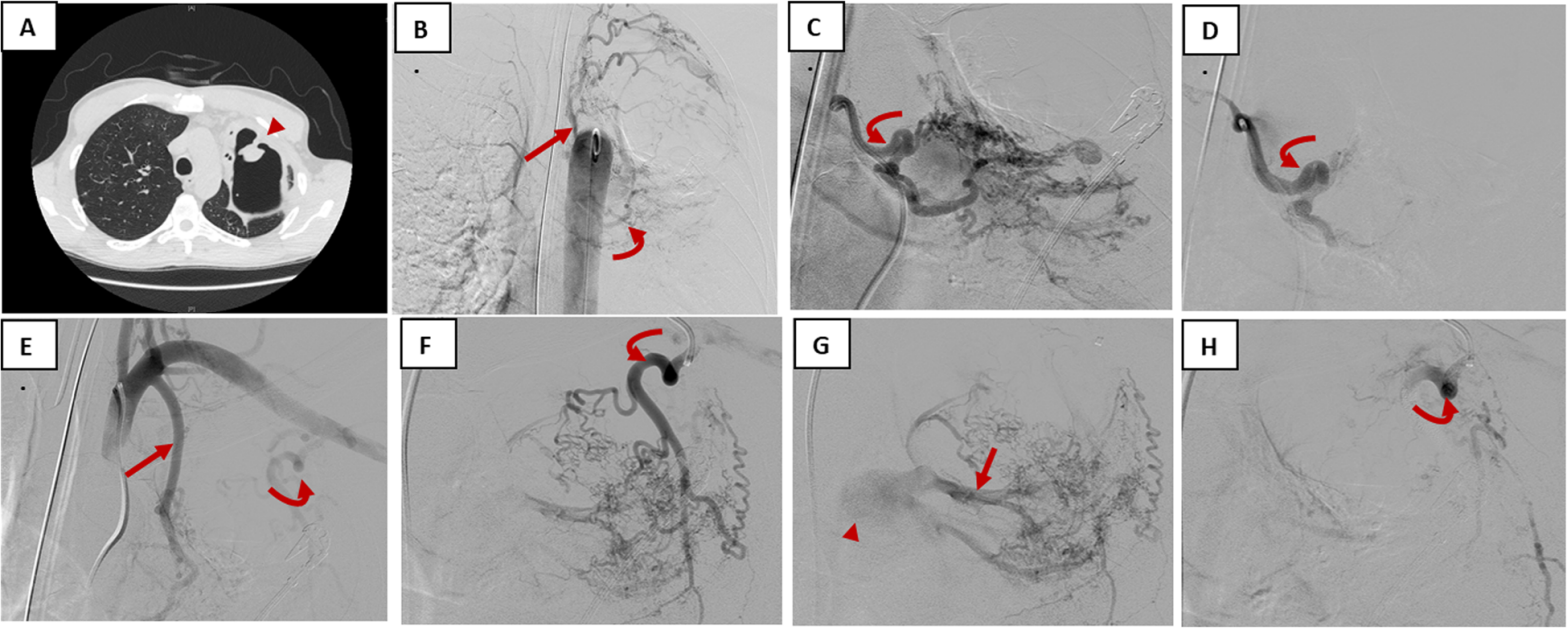
Case presentation. A patient in his late thirties with HIV, tuberculosis (4-drug treatment in 2009), and LUL cavitary lesion with aspergilloma presenting with life-threatening hemoptysis. **a** Single image from axial computed tomography shows aspergilloma (arrowhead), which was the likely etiology of this patient’s hemoptysis. **b** Flush thoracic aortogram demonstrates hypertrophied bronchial arteries (curved arrow) and superior intercostal arteries (straight arrow) supplying the aspergilloma. **c** Representative image of selective angiogram of the larger of the 2 left bronchial arteries shows hypertrophied bronchial artery (curved arrow). **d** Angiogram of the bronchial artery (curved arrow) post-bronchial artery embolization with 300–500 μm particles (trisacryl gelatin microspheres) via selective microcatheter demonstrates lack of blood flow to the area of the aspergilloma. **e** Angiogram of the left subclavian artery to evaluate for non-bronchial systemic collaterals demonstrates abnormal neovascularity filling from the left internal mammary artery (straight arrow) and lateral thoracic artery (curved arrow) on the left side. **f** Representative image of selective angiogram of the lateral thoracic branch on the left. **g** Angiogram shows inflammatory neovascular blush (arrowhead) with shunting to the main pulmonary artery (straight arrow). **h** Post-procedural angiogram with 500–700 and 700–900 μm particles demonstrates successful embolization of the lateral thoracic branch on the left.

A patient in his late thirties with HIV infection and a history of tuberculosis treated 6 years ago with a standard 4-drug tuberculosis regimen for 6 months presented to the emergency room with a complaint of coughing up frank red blood. The patient had noted several days of small-volume hemoptysis and sought medical attention when he coughed up a cup filled with blood (250 cc). His exam was notable for a temperature of 101.9, pulse 149, blood pressure 110/72, respiratory rate 18, and pulse oxygen saturation 94% on room air. He had no oral or nasal sources of bleeding. His cardiac exam was remarkable for tachycardia, and his lung exam was significant for reduced breath sounds overlying the left upper lung. He was intubated for airway control due to ongoing bleeding, ultimately requiring 4 units of packed red blood cells. Chest X-ray showed a cavitary left upper lobe lesion, and CT scan confirmed a large, thick-walled cavitary lesion in the left upper lobe (Fig. 2). Additional CT features included a pedunculated soft tissue mass in the cavity and adjacent pleural reaction. Since CT localized the likely site of bleeding, bronchoscopy was not performed. He underwent angiography with successful embolization of two left bronchial arteries, the left internal mammary artery, and left lateral thoracic artery (Fig. 2).

The patient had recurrent bleeding (20 cc with clots) 1 month after hospital discharge and was managed conservatively with antibiotic therapy. Given the risk of further recurrence, the patient underwent definitive left upper lobe resection. Pathology confirmed the presence of an aspergilloma without evidence of mycobacterial infection. Anti-fungal therapy was not instituted as there was no evidence of angio-invasion on histopathological examination. The patient is doing well 3 years post-operatively.

## Diagnostic modalities

The optimal diagnostic approach to life-threatening hemoptysis has not been determined. Chest radiography (CXR; chest X-ray), computed tomography (CT) and bronchoscopy are the most frequently used modalities to localize the bleeding site and are used alone or in combination depending on institutional practice and availability and patient stability. The published studies comparing the accuracy of each technique are relatively few in number and the diagnostic efficacy is influenced by the underlying cause of hemoptysis.

Revel and colleagues reported that CXR identified the bleeding site in 46% of life-threatening hemoptysis cases, and the underlying bleeding cause in 35% [21]. Studies suggest that CT is superior to CXR for detecting the site of bleeding in life-threatening hemoptysis, with correct localization in 70–88.5% of cases [17, 21]. Imaging techniques, however, may not permit the localization of the bleeding site in patients with pre-existing bilateral lung disease and/or aspiration of blood into non-bleeding segments. Furthermore, CT imaging may not be practical in an unstable patient. Flexible bronchoscopy is a useful diagnostic procedure in situations in which the localization of a side of bleeding is necessary, and the patient is too unstable to undergo diagnostic imaging studies. Bronchoscopy may play a pivotal role with regard to localizing the anatomic site of bleeding, clearing the airway of blood to maintain adequate oxygenation and ventilation, and providing the interventional radiologist with valuable anatomical information.

Several studies have compared CT and bronchoscopy [21–24]. A study comparing CT imaging to bronchoscopy to determine the site and cause of life-threatening hemoptysis found that CT localized the site of bleeding in 70% of patients compared to 73% by bronchoscopy, whereas CT imaging determined the actual cause of bleeding in 77% of cases compared with 8% with bronchoscopy [21]. CT can also demonstrate extrapulmonary causes of hemoptysis such as bleeding from a false aortic aneurysm, or from a pulmonary artery aneurysm or pseudoaneurysm. In a study of 40 patients presenting with hemoptysis who had a normal bronchoscopy, subsequent CT scan detected an etiology for the hemoptysis in 50% [22]. While CT appears to have the highest diagnostic yield for life-threatening hemoptysis, fiberoptic bronchoscopy remains invaluable for patients needing airway control and in patients with bilateral lung disease. In some cases, the combination of bronchoscopy and CT may be more effective than either alone.

## Management

While there are several modalities to treat life-threatening hemoptysis, there are no existing guidelines on how to best manage life-threatening hemoptysis. As outlined below and in the flow diagram (Fig. 1), a systematic and multidisciplinary team approach is required for the management of life-threatening hemoptysis.

### Initial stabilization and airway management

Initial management should focus on airway control, volume resuscitation (if needed), and correction of any bleeding disorder. Patients presenting with life-threatening hemoptysis should be managed in an intensive care setting. Patient positioning can be utilized to minimize aspiration of blood into the unaffected lung until airway control is achieved. If the site of bleeding is known, the patient should be placed in the lateral decubitus position with the bleeding side down. In non-life-threatening hemoptysis, a few studies have reported decrease in hemoptysis and need for interventional procedures with nebulized and intravenous tranexamic acid, a synthetic anti-fibrinolytic agent [25–28]. Although not studied in life-threatening hemoptysis, tranexamic acid can be considered as a temporizing agent prior to definitive intervention.

Intubation for airway control is typically required in life-threatening hemoptysis. Endotracheal intubation is generally accomplished with a single-lumen endotracheal tube size 8.0 mm or greater to permit flexible bronchoscopy, if required [6]. Unilateral intubation of one of the mainstem bronchi can be performed to protect the nonbleeding lung from aspiration. While unilateral intubation does not allow bronchoscopic interventions to the bleeding lung, it can allow effective oxygenation and ventilation while awaiting definitive treatment strategies. Specifically, if the right lung is the site of bleeding, the left mainstem bronchus would be intubated, and if the left lung is bleeding, the right mainstem bronchus would be selectively intubated. It is important to note that a right mainstem intubation carries the risk of right upper lobe occlusion with subsequent atelectasis.

Use of a double-lumen endotracheal tube can provide single lung ventilation and isolation of the bleeding side. However, it is not recommended for routine management of hemoptysis, as it requires highly experienced personnel, is often difficult to place, and the small internal diameters of the bronchial and tracheal limbs can obstruct with blood or clots [10, 29, 30].

Flexible bronchoscopy can help clear the airway of blood to maintain adequate ventilation [7]. Therapeutic techniques to attempt hemostasis via the bronchoscope include cold saline lavage, local instillation of topical vasoconstrictive agents (epinephrine), and balloon blockers [31, 32]. More specialized bronchoscopic techniques for temporary control of bleeding include endobronchial stent tamponade [33], balloon tamponade [34], topical cellulose mesh, or biocompatible glue [6, 35].

### Endovascular treatment

#### Initial angiography and embolization

Angiography is potentially both diagnostic and therapeutic. Following initial stabilization and localization of the bleeding site, first-line therapy for life-threatening hemoptysis is usually bronchial artery embolization (BAE) via the transfemoral approach. Angiographic localization of the bleeding site can be technically challenging, time-consuming, and requires a significant contrast load; therefore, pre-procedural chest CT and/or bronchoscopy to help localize the bleeding site is valuable. Embolization reduces pressure in the abnormal hypertrophic arterial vessels supplying the area of diseased lung, thus decreasing the risk of bleeding. Most interventional radiologists perform a pre-procedural descending thoracic aortogram to identify the origin sites of bronchial arteries from the aorta. Anomalous bronchial arteries as well as non-bronchial systemic arteries supplying the parenchymal abnormality can be visualized on an initial thoracic aortogram in most patients. Bronchial artery catheterization is then performed with selective diagnostic angiographic injections into the arterial circulation harboring the suspected bleeding vessel (e.g., bronchial, intercostal, subclavian, internal mammary arteries).

The findings at angiography warranting BAE include enlarged or tortuous arteries, active contrast extravasation, and hypervascularity. These findings are more common in bleeding due to chronic lung inflammation compared with malignancies [36]. Goh et al reported that 30% of patients with malignancies had no angiographic abnormalities whereas only 5% of patients with TB had no abnormalities [37]. Thus, the determination of which arteries to embolize should be based on a combination of CT (e.g., bronchial artery larger than 2 mm on CT), bronchoscopic location of bleeding (when applicable), and the above angiographic findings. Once the bleeding vessel is identified during angiography, super-selective arterial embolization is performed, most commonly with microparticle embolic agents (e.g., polyvinyl alcohol (PVA) particles or trisacryl gelatin microspheres) [38–40]. Super-selective techniques allow catheterization of smaller, distal, and torturous arteries. The choice of the embolization material is important to the success of the intervention and is dependent upon the size and site of the bleeding vessel, the ease of access and deployment of the occlusive material to the vessel, the size of the catheter being used, the durability of occlusion as well as the tendency for recanalization [38].

If the interventional radiologist is unable to localize the site of bleeding to the bronchial circulation, the non-bronchial systemic and pulmonary circulations can be sequentially evaluated for bleeding. Studies suggest that systematically searching for non-bronchial systemic collaterals reduces rates of recurrence and leads to better overall hemoptysis control. Many interventional radiologists, therefore, recommend actively searching for and embolizing as many non-bronchial systemic collaterals in the first BAE procedure itself to decrease recurrence rates [14]. Lastly, pulmonary artery angiography is sometimes performed to assess for pulmonary artery abnormalities such as a Rasmussen aneurysm.

#### Complications

The most common complication of BAE is self-limited chest pain which occurs in 1.4–34.5% of patients [36, 41, 42]. Transient dysphagia may occur in up to 30% of procedures as a result of esophageal “nontarget” embolization [36, 41, 42]. This is also usually a self-resolving adverse event [36]. Other procedural complications include groin hematoma, subintimal dissection, or perforation of the arteries by the guidewire, cortical blindness caused by embolization of the occipital cortex, bronchial stenosis, necrosis, bronchoesophageal fistula, pulmonary infarction, and ischemic colitis [36, 38, 42]. The most serious complication is unintended spinal artery embolization leading to transient or persistent paraparesis or paraplegia [36]. This complication is attributed to inadvertent embolization of the spinal artery which originates from a bronchial artery in 5% of patients. While the reported rate of paraparesis or paraplegia is 0.6–4.4%, the higher numbers reflect procedures performed prior 2010 when super-selective microcatheter techniques, which allow bypassing of the spinal arteries, were introduced. This technique enables the distal cannulation of the target vessel beyond the origin of spinal branches to minimize neurological complications.

#### Rebleeding after embolization therapy

A systematic review reported that the immediate clinical success of BAE (defined as the cessation of bleeding within 24 h of BAE or within the same admission) is 70–99% [36]. Hemoptysis recurrence rate (defined in this study as significant hemoptysis occurring after discharge, requiring either hospital admission, medical management, or repeat intervention) following BAE is in the range of 9.8–57.5% with the median time until recurrent bleed between 6 months to 1 year [36]. Angiographic features associated with high recurrence rates include (1) the presence of non-bronchial systemic collaterals, (2) bronchopulmonary shunting, and (3) incomplete initial embolization [36, 43, 44]. Recurrence of bleeding within 2 weeks after bronchial artery embolization is usually due to incomplete embolization due to lack of a complete search of all offending vessels or inability to embolize all arteries, including the non-bronchial systemic artery collaterals [14]. The second peak for recurrence of bleeding is from 1-2 years after the initial embolization. The processes contributing to recurrent bleeding include recanalization of the embolized vessel, non-bronchial systemic arterial supply, and/or progression of the causative underlying lung disease [45, 46]. Furthermore, certain disease entities, such as sarcoidosis, aspergilloma, and sequela of tuberculosis, confer a high risk of recurrent bleeding and may require more definitive long-term management after initial stabilization.

A study from the USA describing an 11-year experience of 69 patients who underwent 97 BAE procedures, reported that recurrent bleeding and mortality [hazard ratio for death of 4 (95% CI 2.6–14.6)] were increased among patients with sarcoidosis compared with other causes [44]. The median time to recurrent bleeding following BAE was 29 days among patients with sarcoidosis compared with 293 days among patients without sarcoidosis.

Several studies report a high bleeding recurrence rate following BAE in patients with aspergillomas, cystic fibrosis, and TB [43, 46–49]. Life-threatening hemoptysis in patients with aspergillomas has been associated with a 25-30% mortality rate. The majority of patients with aspergillomas will rebleed over time if they are not treated surgically. BAE is therefore a temporary treatment strategy for life-threatening hemoptysis in such patients. In patients with cystic fibrosis, rebleeding occurs in approximately 30 to 40 percent of cases [48, 49]. TB and TB sequelae increase the risk of recurrent life-threatening hemoptysis following BAE [43, 46, 50]. In addition, reactivation TB and multidrug-resistant (MDR) TB are considered risk factors for recurrent bleeding [43, 47]. In areas with a high prevalence of hemoptysis due to tuberculosis, a risk score that identifies patients at greater risk of rebleeding may be useful [46, 51].

Repeat embolization is an appropriate treatment approach for recurrent hemoptysis from all etiologies. As an example, Lee et al showed that repeat BAE achieves comparable immediate and clinical success and similar recurrence rates when compared with the initial embolization procedure [52]. The outcomes of repeat arterial embolization are generally favorable in terms of recurrent bleeding within 2 weeks after bronchial artery embolization [45]. While repeat BAE can help control recurrent hemoptysis, definitive surgery is often needed for hemoptysis refractory to multiple repeat embolization.

### Bronchoscopic therapies

More definitive endoluminal techniques may be useful when the source of bleeding is endobronchial, when the source is not amenable to bronchial artery embolization (BAE), or when BAE has failed. Possible techniques include local laser therapies such as Nd:YAG laser or argon plasma coagulation of the bleeding mucosa with the goal of hemostasis [53–55]. Bronchoscopic laser therapy cannot be used in patients requiring a fraction of inspired oxygen (FIO2) > 0.40 because of the risk of airway fire [56].

Some techniques have a delayed onset of action and are more useful to prevent future bleeding. As an example, cryotherapy has no role in the management of life-threatening hemoptysis due to its delayed onset of effect. Similarly, brachytherapy is not an immediate treatment option for life-threatening bleeding [6].

Rigid bronchoscopy can be safer and more efficient than flexible bronchoscopy for controlling life-threatening hemoptysis [57]; however, rapid availability varies from institution to institution. Rigid bronchoscopy allows for more efficient suctioning of blood clots which leads to better airway visualization [30, 58]. Bronchial blockers can be used via rigid or flexible bronchoscopy to tamponade bleeding sites and preventing spillage of blood into the non-bleeding lung. Bronchial blockers may also achieve stable one-lung ventilation until definitive treatment is offered. Endoscopic therapies such as laser, electrocautery, and argon plasma coagulation can be safely performed through a rigid bronchoscope to control bleeding from visualized endobronchial lesions [30].

### Timing and role of surgery in the management of hemoptysis

Due to the availability of safe and effective endovascular embolization techniques, BAE has largely replaced emergent surgery for the management of life-threatening hemoptysis. In a study by Sopko et al., emergent lung resection for life-threatening hemoptysis had a reported mortality rate of 40%, compared with an 18% mortality rate when performed electively [38]. In a separate study, when surgical management of life-threatening hemoptysis was avoided in the first 48 h and conducted only after technical failure or early relapse following BAE, lower in-hospital mortality and surgical morbidity rates were observed in comparison with earlier studies favoring surgery as first-line therapy [59]. Similarly, Andréjak et al. reported in 111 lung resections for severe hemoptysis, mortality rates were 35% when performed emergently compared with 0% in patients undergoing resection electively after hospital discharge [60]. Thus, emergent surgical intervention to manage life-threatening hemoptysis is reserved for traumatic injury to the chest, iatrogenic pulmonary artery rupture, or pulmonary artery hemorrhage in the context of a resectable lung tumor.

The specific indications for surgical intervention in the setting of life-threatening hemoptysis include the technical failure of the BAE procedure, recurrent hemoptysis despite multiple BAE interventions, and maximum medical therapy, or life-threatening circumstances during the hemoptysis episode that does not permit the safe performance of an interventional radiologic procedure [6, 38]. Lastly, consideration of definitive surgery may be particularly important in patients with aspergillomas and in those patients with TB who have high risk scores for rebleeding after BAE.

## Conclusion

The diagnostic evaluation of patients with life-threatening hemoptysis remains focused on the localization of the bleeding site and underlying cause, which can be performed quickly with CT imaging in patients who have adequate oxygenation, ventilation, and are hemodynamically stable. Bronchoscopy remains invaluable for patients needing airway control and those in whom CT imaging cannot localize the bleeding site (e.g., bilateral lung disease). The more widespread availability of bronchial artery embolization has led to a shift in the management of life-threatening hemoptysis. BAE, rather than surgical resection, has become the primary intervention to control bleeding in the acute setting. Recurrent bleeding following BAE remains high. Repeat BAE, often multiple times, is appropriate for all etiologies of recurrent bleeding. Patients, especially those with aspergillomas and certain TB patients with high-risk for rebleeding, should be evaluated for elective surgical intervention following hospital discharge.

## Data Availability

N/A

## Abbreviations

BAE: Bronchial artery embolization
CT: Computerized tomography
CXR: Chest X-ray
FIO2: Fraction of inspired oxygen
Nd:YAG: Neodymium-yttrium-aluminum-garnet

## References

[1] Hirshberg B, Biran I, Glazer M, Kramer MR (1997). Hemoptysis: etiology, evaluation, and outcome in a tertiary referral hospital. Chest..

[2] Corey R, Hla KM (1987). Major and massive hemoptysis: reassessment of conservative management. Am J Med Sci..

[3] Garzon AA, Gourin A (1978). Surgical management of massive hemoptysis. A ten-year experience. Ann Surg..

[4] Knott-Craig CJ, Oostuizen JG, Rossouw G, Joubert JR, Barnard PM (1993). Management and prognosis of massive hemoptysis. Recent experience with 120 patients. J Thorac Cardiovasc Surg..

[5] Mal H, Rullon I, Mellot F, Brugière O, Sleiman C, Menu Y (1999). Immediate and long-term results of bronchial artery embolization for life-threatening hemoptysis. Chest..

[6] Sakr L, Dutau H (2010). Massive hemoptysis: an update on the role of bronchoscopy in diagnosis and management. Respiration..

[7] Dweik RA, Stoller JK (1999). Role of bronchoscopy in massive hemoptysis. Clin Chest Med..

[8] Fartoukh M, Khoshnood B, Parrot A, Khalil A, Carette MF, Stoclin A (2012). Early prediction of in-hospital mortality of patients with hemoptysis: an approach to defining severe hemoptysis. Respiration..

[9] Porter DK, Van Every MJ, Anthracite RF, Mack JW (1983). Massive hemoptysis in cystic fibrosis. Arch Intern Med..

[10] Cahill BC, Ingbar DH (1994). Massive hemoptysis. Assessment and management. Clin Chest Med.

[11] Santiago S, Tobias J, Williams AJ (1991). A reappraisal of the causes of hemoptysis. Arch Intern Med..

[12] Johnston H, Reisz G (1989). Changing spectrum of hemoptysis. Underlying causes in 148 patients undergoing diagnostic flexible fiberoptic bronchoscopy. Arch Intern Med..

[13] Mondoni M, Carlucci P, Job S, Parazzini EM, Cipolla G, Pagani M (2018). Observational, multicentre study on the epidemiology of haemoptysis. Eur Respir J.

[14] Yoon W, Kim JK, Kim YH, Chung TW, Kang HK (2002). Bronchial and nonbronchial systemic artery embolization for life-threatening hemoptysis: a comprehensive review. Radiographics..

[15] Deffebach ME, Charan NB, Lakshminarayan S, Butler J (1987). The bronchial circulation. Small, but a vital attribute of the lung. Am Rev Respir Dis..

[16] Larici AR, Franchi P, Occhipinti M, Contegiacomo A, del Ciello A, Calandriello L (2014). Diagnosis and management of hemoptysis. Diagn Interv Radiol..

[17] Khalil A, Fedida B, Parrot A, Haddad S, Fartoukh M, Carette MF (2015). Severe hemoptysis: from diagnosis to embolization. Diagn Interv Imaging..

[18] Jean-Baptiste E (2001). Clinical assessment and management of massive hemoptysis. Crit Care Med..

[19] Remy-Jardin M, Bouaziz N, Dumont P, Brillet PY, Bruzzi J, Remy J (2004). Bronchial and nonbronchial systemic arteries at multi-detector row CT angiography: comparison with conventional angiography. Radiology..

[20] Rémy-Jardin M, Rémy J (1990). The non-bronchial systemic vascular circulation of the lung. Rev Mal Respir..

[21] Revel MP, Fournier LS, Hennebicque AS, Cuenod CA, Meyer G, Reynaud P (2002). Can CT replace bronchoscopy in the detection of the site and cause of bleeding in patients with large or massive hemoptysis?. AJR Am J Roentgenol..

[22] Millar AB, Boothroyd AE, Edwards D, Hetzel MR (1992). The role of computed tomography (CT) in the investigation of unexplained haemoptysis. Respir Med..

[23] Naidich DP, Funt S, Ettenger NA, Arranda C (1990). Hemoptysis: CT-bronchoscopic correlations in 58 cases. Radiology..

[24] Set PA, Flower CD, Smith IE, Chan AP, Twentyman OP, Shneerson JM (1993). Hemoptysis: comparative study of the role of CT and fiberoptic bronchoscopy. Radiology..

[25] Hankerson MJ, Raffetto B, Mallon WK, Shoenberger JM (2015). Nebulized tranexamic acid as a noninvasive therapy for cancer-related hemoptysis. J Palliat Med..

[26] Komura S, Rodriguez RM, Peabody CR (2018). Hemoptysis? Try inhaled tranexamic acid. J Emerg Med..

[27] Bellam BL, Dhibar DP, Suri V, Sharma N, Varma SC, Malhotra S (2016). Efficacy of tranexamic acid in haemoptysis: a randomized, controlled pilot study. Pulm Pharmacol Ther..

[28] Wand O, Guber E, Guber A, Epstein Shochet G, Israeli-Shani L, Shitrit D (2018). Inhaled tranexamic acid for hemoptysis treatment: a randomized controlled trial. Chest..

[29] Santana-Cabrera L, Arroyo MF, Rodriguez AU, Sanchez-Palacios M (2010). Double-lumen endobronchial tube in the emergency management of massive hemoptysis. J Emerg Trauma Shock..

[30] Radchenko C, Alraiyes AH, Shojaee S (2017). A systematic approach to the management of massive hemoptysis. J Thorac Dis..

[31] Caddell B, Yelverton B, Tippett JC, Ravi Y, Sai-Sudhakar CB, Culp WC (2017). Management of massive hemoptysis after pulmonary thromboembolectomy using a double bronchial blocker system. J Cardiothorac Vasc Anesth..

[32] Dutau H, Palot A, Haas A, Decamps I, Durieux O (2006). Endobronchial embolization with a silicone spigot as a temporary treatment for massive hemoptysis: a new bronchoscopic approach of the disease. Respiration..

[33] Barisione E, Genova C, Grosso M, Pasquali M, Blanco A, Felletti R (2017). Palliative treatment of life-threatening hemoptysis with silicone stent insertion in advanced lung cancer. Monaldi Arch Chest Dis..

[34] Correia S, Dionísio J (2014). Duro da Costa JJ. Modified technique of endobronchial balloon tamponade for persistent hemoptysis. J Bronchology Interv Pulmonol..

[35] Peralta AR, Chawla M, Lee RP (2018). Novel bronchoscopic management of airway bleeding with absorbable gelatin and thrombin slurry. J Bronchology Interv Pulmonol..

[36] Panda A, Bhalla AS, Goyal A (2017). bronchial artery embolization in hemoptysis: a systematic review. Diagn Interv Radiol..

[37] Yu-Tang Goh P, Lin M, Teo N, En Shen Wong D (2002). Embolization for hemoptysis: a six -year review. Cardiovasc Intervent Radiol..

[38] Sopko DR, Smith TP (2011). Bronchial artery embolization for hemoptysis. Semin Intervent Radiol..

[39] Ittrich H, Klose H, Adam G (2015). Radiologic management of haemoptysis: diagnostic and interventional bronchial arterial embolisation. Rofo..

[40] Vaidya S, Tozer KR, Chen J (2008). An overview of embolic agents. Semin Intervent Radiol..

[41] Fruchter O, Schneer S, Rusanov V, Belenky A, Kramer MR (2015). Bronchial artery embolization for massive hemoptysis: long-term follow-up. Asian Cardiovasc Thorac Ann..

[42] Anuradha C, Shyamkumar NK, Vinu M, Babu NR, Christopher DJ (2012). Outcomes of bronchial artery embolization for life-threatening hemoptysis due to tuberculosis and post-tuberculosis sequelae. Diagn Interv Radiol..

[43] Hwang HG, Lee HS, Choi JS, Seo KH, Kim YH, Na JO (2013). Risk factors influencing rebleeding after bronchial artery embolization on the management of hemoptysis associated with pulmonary tuberculosis. Tuberc Respir Dis (Seoul)..

[44] Tom LM, Palevsky HI, Holsclaw DS, Trerotola SO, Dagli M, Mondschein JI (2015). Recurrent bleeding, survival, and longitudinal pulmonary function following bronchial artery embolization for hemoptysis in a U.S. adult population. J Vasc Interv Radiol.

[45] Hayakawa K, Tanaka F, Torizuka T, Mitsumori M, Okuno Y, Matsui A (1992). Bronchial artery embolization for hemoptysis: immediate and long-term results. Cardiovasc Intervent Radiol..

[46] van den Heuvel MM, Els Z, Koegelenberg CF, Naidu KM, Bolliger CT, Diacon AH (2007). Risk factors for recurrence of haemoptysis following bronchial artery embolisation for life-threatening haemoptysis. Int J Tuberc Lung Dis..

[47] Shin BS, Jeon GS, Lee SA, Park MH (2011). Bronchial artery embolisation for the management of haemoptysis in patients with pulmonary tuberculosis. Int J Tuberc Lung Dis..

[48] Roebuck DJ, Barnacle AM (2008). Haemoptysis and bronchial artery embolization in children. Paediatr Respir Rev..

[49] Swanson KL, Johnson CM, Prakash UB, McKusick MA, Andrews JC, Stanson AW (2002). Bronchial artery embolization: experience with 54 patients. Chest..

[50] Kim SW, Lee SJ, Ryu YJ, Lee JH, Chang JH, Shim SS (2015). Prognosis and predictors of rebleeding after bronchial artery embolization in patients with active or inactive pulmonary tuberculosis. Lung..

[51] Gross AM, Diacon AH, van den Heuvel MM, Janse van Rensburg J, van Rensburg J, Harris D (2009). Management of life-threatening haemoptysis in an area of high tuberculosis incidence. Int J Tuberc Lung Dis..

[52] Lee S, Chan JW, Chan SC, Chan YH, Kwan TL, Chan MK (2008). Bronchial artery embolisation can be equally safe and effective in the management of chronic recurrent haemoptysis. Hong Kong Med J..

[53] Cho YJ, Murgu SD, Colt HG (2007). Bronchoscopy for bevacizumab-related hemoptysis. Lung Cancer..

[54] Dabó H, Gomes R, Teixeira N, Teixeira G, Fernandes G, Magalhães A (2016). Tracheal lobular capillary hemangioma treated with laser photocoagulation. J Bras Pneumol..

[55] Han CC, Prasetyo D, Wright GM (2007). Endobronchial palliation using Nd:YAG laser is associated with improved survival when combined with multimodal adjuvant treatments. J Thorac Oncol..

[56] Dalar L, Sökücü SN, Özdemir C, Büyükkale S, Altın S (2015). Endobronchial argon plasma coagulation for treatment of Dieulafoy disease. Respir Care..

[57] Colchen A, Fischler M (2011). Emergency interventional bronchoscopies. Rev Pneumol Clin..

[58] Alraiyes AH, Machuzak MS (2014). Rigid bronchoscopy. Semin Respir Crit Care Med..

[59] Shigemura N, Wan IY, Yu SC, Wong RH, Hsin MK, Thung HK (2009). Multidisciplinary management of life-threatening massive hemoptysis: a 10-year experience. Ann Thorac Surg..

[60] Andréjak C, Parrot A, Bazelly B, Ancel PY, Djibré M, Khalil A (2009). Surgical lung resection for severe hemoptysis. Ann Thorac Surg..

